# Adaptive Guided Equilibrium Optimizer with Spiral Search Mechanism to Solve Global Optimization Problems

**DOI:** 10.3390/biomimetics8050383

**Published:** 2023-08-23

**Authors:** Hongwei Ding, Yuting Liu, Zongshan Wang, Gushen Jin, Peng Hu, Gaurav Dhiman

**Affiliations:** 1 School of Information Science and Engineering, Yunnan University, Kunming 650106, China; wzs_ynu@163.com (H.D.); ytliu@mail.ynu.edu.cn (Y.L.); 2 Glasgow College, University of Electronic Science and Technology of China, Chengdu 611731, China; 12020215187@mail.ynu.edu.cn; 3 Research and Development Department, Youbei Technology Co., Ltd., Kunming 650011, China; hupeng@ubeisci.com; 4 Department of Electrical and Computer Engineering, Lebanese American University, Byblos P.O. Box 13-5053, Lebanon; gdhiman0001@163.com

**Keywords:** equilibrium optimizer, metaheuristics, global optimization, nature-inspired, mobile robot path planning

## Abstract

The equilibrium optimizer (EO) is a recently developed physics-based optimization technique for complex optimization problems. Although the algorithm shows excellent exploitation capability, it still has some drawbacks, such as the tendency to fall into local optima and poor population diversity. To address these shortcomings, an enhanced EO algorithm is proposed in this paper. First, a spiral search mechanism is introduced to guide the particles to more promising search regions. Then, a new inertia weight factor is employed to mitigate the oscillation phenomena of particles. To evaluate the effectiveness of the proposed algorithm, it has been tested on the CEC2017 test suite and the mobile robot path planning (MRPP) problem and compared with some advanced metaheuristic techniques. The experimental results demonstrate that our improved EO algorithm outperforms the comparison methods in solving both numerical optimization problems and practical problems. Overall, the developed EO variant has good robustness and stability and can be considered as a promising optimization tool.

## 1. Introduction

Optimization problems have gained significant attention in engineering and scientific domains. In general, the objective of optimization problems is to achieve the best possible outcome by minimizing the corresponding objective function while minimizing undesirable factors [1]. These problems may involve constraints, which means that various constraints need to be satisfied during the optimization process. Based on their characteristics, optimization problems can be classified into two categories: local optimization and global optimization. Local optimization aims to determine the optimal value within a local region [2]. On the other hand, global optimization aims to find the optimal value within a given region. Therefore, global optimization is more challenging compared to local optimization.

To address various types of global optimization problems, numerous optimization techniques have been developed [3]. Among the current optimization techniques, metaheuristic algorithms have gained widespread attention due to their advantages of being gradient-free, not requiring prior information about the problem, and offering high flexibility. Metaheuristic algorithms provide acceptable solutions with relatively fewer computational costs [4]. Based on their sources of inspiration, metaheuristic algorithms can be classified into four categories: swarm intelligence algorithms, evolutionary optimization algorithms, physics-inspired algorithms, and human-inspired algorithms [5,6]. Swarm intelligence optimization algorithms simulate the cooperative behavior observed in animal populations in nature. Examples of such algorithms include Artificial Bee Colony (ABC) [7], Particle Swarm Optimization (PSO) [8], Grey Wolf Optimization (GWO) [9], Firefly Optimization (FA) [10], Ant Colony Optimization (ACO) [11], Harris Hawks Optimization Algorithm (HHO) [12], Salp Swarm Algorithm (SSA) [13], and others. The second category draws inspiration from the concept of natural evolution. These algorithms include, but are not limited to, Evolution Strategy (ES) [14], Differential Evolution (DE) [15], Backtracking Search Algorithm (BSA) [16], Stochastic Fractal Search (SFS) [17], Wildebeests Herd Optimization (WHO) [18]. The third class of metaheuristic algorithms is inspired by physics concepts. The following algorithms are some examples of physics-inspired algorithms: Simulated Annealing (SA) [19] algorithm, Big Bang-Big Crunch (BB-BC) [20] algorithm, Central Force Optimization (CFO) [21], Intelligent Water Drops (IWD) [22], Slime Mold Algorithm (SMA) [23], Gravitational Search Algorithm (GSA) [24], Black Hole Algorithm (BHA) [25], Water Cycle Algorithm (WCA) [26], Lightning Search Algorithm (LSA) [27]. As these physics-inspired algorithms proved to be effective in engineering and science, more similar algorithms were developed, such as Multi-Verse Optimizer (MVO) [28], Thermal Exchange Optimization (TEO) [29], Henry Gas Solubility Optimization (HGSO) [30], Equilibrium Optimizer (EO) [31], Archimedes Optimization Algorithm (AOA) [32], and Special Relativity Search (SRS) [33]. The last class of metaheuristic techniques simulates human behavior, such as Seeker Optimization Algorithm (SOA) [34], Imperialist Competitive Algorithm (ICA) [35], Brain Storm Optimization (BSO) [36], and Teaching-Learning-Based Optimization (TLBO) [37].

The most popular categories among these are swarm intelligence algorithms and physics-inspired algorithms, as they offer reliable metaphors and simple yet efficient search mechanisms. In this work, we consider leveraging the search behavior of swarm intelligence algorithms to enhance the performance of a physics-inspired algorithm called EO. EO simulates the dynamic equilibrium concept of mass in physics. In a container, the attempt to achieve dynamic equilibrium of mass within a controlled volume is performed by expelling or absorbing particles, which are referred to as a set of operators employed during the search in the solution space. Based on these search models, EO has demonstrated its performance across a range of real-world problems, such as solar photovoltaic parameter estimation [38], feature selection [39], multi-level threshold image segmentation [40], and so on. Despite the simple search mechanism and effective search capability of the EO algorithm, it still suffers from limitations, such as falling into local optima traps and imbalanced exploration and exploitation. To address these limitations, this paper proposes a novel variant of EO called SSEO by introducing an adaptive inertia weight factor and a swarm-based spiral search mechanism. The adaptive inertia weight factor is employed to enhance population diversity and strengthen the algorithm’s global exploration ability, while the spiral search mechanism is introduced to expand the search space of particles. These two mechanisms work synergistically to achieve a balance between exploration and exploitation phases of the algorithm. To evaluate the performance of the proposed algorithm, 29 benchmark test functions from the IEEE CEC 2017 are used. The results obtained by SSEO are compared against several state-of-the-art metaheuristic algorithms, including the basic EO, spiral search mechanism-based metaheuristics, and recently proposed variants of EO. The test results demonstrate that SSEO provides competitive results on almost all functions compared to the benchmark algorithms. Additionally, the SSEO algorithm is tested on a real-world problem of MRPP and compared against several classical metaheuristic algorithms. Simulation results on three maps with different characteristics indicate that the developed SSEO-based path planning approach can find obstacle-free paths with smaller computational costs, suggesting its promising potential as a path planner. The main contributions of this work can be summarized as follows:

1. Utilizing the structure of EO, an enhanced variant called SSEO is proposed, which employs two simple yet effective mechanisms to improve population diversity, convergence performance, and the balance between exploration and exploitation.
2. SSEO incorporates an adaptive inertia weight mechanism to enhance population diversity in EO and a swarm-inspired spiral search mechanism to expand the search space. The simultaneous operation of these two mechanisms ensures a stable balance between exploration and exploitation.
3. To evaluate the effectiveness and problem-solving capability of SSEO, the CEC 2017 benchmark function set is utilized. Experimental results demonstrate that the proposed algorithm outperforms the basic EO, several recently reported EO variants, and other state-of-the-art metaheuristic algorithms.
4. To investigate the ability of the proposed EO variant in solving real-world problems, it is applied to address the MRPP problem. Simulation results indicate that, compared to the benchmark algorithms, SSEO can provide reasonable collision-free paths for the mobile robot in different environmental settings.

The remaining parts of this paper are organized as follows: Section 2 provides a literature review. Section 3 introduces the search framework and mathematical model of the basic EO. Section 4 reports the developed strategies and the framework of the SSEO algorithm. The validation of the SSEO algorithm’s effectiveness using CEC 2017 functions is presented in Section 5. Section 6 introduces the developed SSEO-based MRPP approach and validates its performance. Finally, Section 7 summarizes the research and extends future research directions.

## 2. Related Work

Well-established metaheuristic algorithms are equipped with reasonable mechanisms to transition between exploration and exploitation. Global exploration allows the algorithm to comprehensively search the solution space and explore unknown regions, while local exploitation aids in fine-tuning solutions within specific areas to improve solution accuracy. EO algorithm, a recently proposed physics-inspired metaheuristic algorithm, is based on metaphors from the field of physics. The efficiency and applicability of EO have been demonstrated in benchmark function optimization problems as well as real-world problems. However, despite EO’s attempt to design effective search models based on reliable metaphors, the transition from exploration to exploitation during the search process is still imperfect, resulting in limitations such as getting trapped in local optima and premature convergence.

To mitigate the inherent limitations of EO and provide a viable alternative efficient optimization tool for the optimization community, many researchers have made improvements and proposed different versions of EO variants. Gupta et al. [41] introduced mutation strategies and additional search operators, referred to as mEO, into the basic EO. The mutation operation is used to overcome the problem of population diversity loss during the search process, and the additional search operators assist the population in escaping local optima. The performance of mEO was tested on 33 commonly used benchmark functions and four engineering design problems. Experimental results demonstrated that mEO effectively enhances the search capability of the EO algorithm.

Houssein et al. [42] strengthened the balance between exploration and exploitation in the basic EO algorithm by employing the dimension hunting technique. The performance of the proposed EO variant was tested using the CEC 2020 benchmark test suite and compared with advanced metaheuristic methods. Comparative results showed the superiority of the proposed approach. Additionally, the proposed EO variant was applied to multi-level thresholding image segmentation of CT images. Comparative results with a set of popular image segmentation tools showed good performance in terms of segmentation accuracy.

Liu et al. [43] introduced three new strategies into EO to improve algorithm performance. In this version of EO, Levy flight was used to enhance particle search in unknown regions, the WOA search mechanism was employed to strengthen local exploitation tendencies, and the adaptive perturbation technique was utilized to enhance the algorithm’s ability to avoid local optima. The performance of the algorithm was tested on the CEC 2014 benchmark test suite and compared with several well-known algorithms. Comparative results showed that the proposed EO variant outperformed the compared algorithms in the majority of cases. Furthermore, the algorithm’s capability to solve real-world problems was investigated using engineering design cases, demonstrating its practicality in addressing real-world problems.

Tan et al. [44] proposed a hybrid algorithm called EWOA, which combines EO and WOA, aiming to compensate for the inherent limitations of the EO algorithm. Comparative results with the basic EO, WOA, and several classical metaheuristic algorithms showed that EWOA mitigates the tendency of the basic EO algorithm to get trapped in local optima to a certain extent.

Zhang et al. [45] introduced an improved EO algorithm, named ISEO, by incorporating an information exchange reinforcement mechanism to overcome the weak inter-particle information exchange capability in the basic EO. In ISEO, a global best-guided mechanism was employed to enhance the guidance towards a balanced state, a reverse learning technique was utilized to assist the population in escaping local optima, and a differential mutation mechanism was expected to improve inter-particle information exchange. These three mechanisms were simultaneously embedded in EO, resulting in an overall improved algorithm performance. The effectiveness of ISEO was demonstrated on a large number of benchmark test functions and engineering design cases.

Minocha et al. [46] proposed an EO variant called MEO, which enhances the convergence performance of the basic EO. In MEO, adjustments were made to the construction of the balance pool to strengthen the algorithm’s search intensity, and the Levy flight technique was introduced to improve global search capability. To investigate the convergence performance of MEO, 62 benchmark functions with different characteristics and five engineering design cases were utilized. Experimental results demonstrated that MEO provides excellent robustness and convergence compared to other algorithms.

Balakrishnan et al. [47] introduced an improved version of EO, called LEO, for feature selection problems. LEO inherits the framework and Levy flight mechanism of EO with the expectation of providing a better search capability in comparison to the basic EO. To validate the performance of LEO, the algorithm was tested on a microarray cancer dataset and compared with several high-performing feature selection methods. Comparative results showed significant advantages of LEO in terms of accuracy and speed compared to the compared algorithms.

## 3. The Original EO

EO is a recently proposed novel physics-based metaheuristic algorithm designed for addressing global optimization problems. In the initial stage, EO randomly generates a set of particles to initiate the optimization process. In EO, the concept of concentration is used to represent the state of particles, similar to the particle positions in PSO. The algorithm expects the particles to achieve a state of balance within a mass volume, and the process of striving towards this balance state constitutes the optimization process of the algorithm, with the final balanced state being the optimal solution discovered by the algorithm. The EO algorithm generates the initial population in the following manner:

(1) $$C_{i}^{initial}=C_{min}+rand_{i}\left(C_{max}-C_{min}\right)i=1,2,\ldots \ldots ,N$$

where $rand_{i}$ is a random vector between [0, 1], $C_{max}$ and $C_{min}$ are the boundaries of the search region, and *N* is the population size.

After the search process is started, the initial particles are updated in concentration according to the following equation:

(2) $$C=C_{eq}+\left(C-C_{eq}\right)\cdot F+\frac{G}{\lambda V}\left(1-F\right)$$

where $C_{eq}$ is the concentration of a randomly selected particle in the equilibrium pool; *F* represents the exponent term, responsible for adjusting the global and local search behavior; *G* represents the generation rate, responsible for local search; λ is a random value; and *V* is a constant with a value of 1. The second term in the equation is responsible for global exploration, while the third term is responsible for local exploitation. The equilibrium pool is constructed as follows:

(3) $$C_{eq,pool}=\left\{\left.C_{eq(1)},C_{eq(2)},C_{eq(3)},C_{eq(4)},C_{eq(ave)}\right\}\right.$$

where $C_{eq(1)}$, $C_{eq(2)}$, $C_{eq(3)}$, and $C_{eq(4)}$ are the four particles with the optimal concentration in the population, which are called equilibrium candidates, and $C_{eq(ave)}$ is the mean value of the above four particles. In the optimization process, there is a lack of information about the equilibrium state, and the equilibrium candidates are used to act as equilibrium states to drive the optimization process.

The concept of exponential term is used in EO to adjust the global search and local search behavior, and the mathematical model of the exponential term *F* is calculated according to the following equation:

(4) $$F=e^{-\lambda (t-t_{0})}$$

where *t* is a nonlinear function of the number of iterations, and $t_{0}$ is a parameter that adjusts the local and global search capabilities of the algorithm. *t* and $t_{0}$ are calculated according to the following equation, respectively.

(5) $$t={\left(1-\frac{Iter}{Max\_iter}\right)}^{\left(a_{2}\frac{Iter}{Max\_iter}\right)}$$

(6) $$t_{0}=\frac{1}{\lambda }\ln\left(-a_{1}sign\left(r-0.5\right)\left(1-e^{\lambda t}\right)\right)+t$$

In Equation (5), Iter and Max_iter denote the current iteration round and the set maximum number of iterations, respectively, and the parameter $a_{2}$ is responsible for adjusting the local exploration capacity of the algorithm and is set as a constant. In Equation (6), the parameter $a_{1}$ is responsible for managing the global exploration capacity of the algorithm and is set as a constant. In the basic EO, $a_{1}$ and $a_{2}$ are set to 0 and 1, respectively. In addition, *r* and λ are random vectors between [0, 1]. Correspondingly, sign(r−0.5) controls the direction of particle concentration change.

The generation rate *G* is the key factor, which is used to fine tune the given region and improve the solution accuracy. *G* is calculated according to the following equation:

(7) $$G=G_{0}e^{-\lambda (t-t_{0})}=G_{0}F$$

where *F* is the exponential term, which is calculated according to Equation (4), and $G_{0}$ is the initial value, which is calculated according to the following equation.

(8) $$G_{0}=GCP\left(C_{eq}-\lambda C\right)$$

(9) $$GCP=\left\{\begin{array}{c} 0.5r_{1}r_{2}\geq GP\\ 0r_{2}<GP \end{array}\right.$$

where $r_{1}$ and $r_{2}$ are random values between [0, 1], and the generation rate control factor GCP controls whether the generation rate can participate in the concentration update process.

From Equation (2), it can be seen that the concentration update mechanism consists of three terms. The first term is the balance candidate to guide the particle update; the second and third terms are the concentration variables, which are responsible for local exploitation and global exploration, respectively. the EO algorithm, with the help of these three behaviors, is able to achieve local exploitation in the early stage and global exploration in the later stage.

## 4. Proposed Improved EO

The effectiveness of the EO algorithm has been demonstrated in numerical optimization problems, engineering design cases, and real-world scenarios, attracting numerous researchers to apply this algorithm to solve problems in their respective fields. However, when faced with challenging optimization tasks, EO still exhibits insufficient exploration capabilities and can get trapped in local optima. To mitigate these limitations, this paper proposes two customized strategies that are embedded into the basic EO algorithm, aiming to develop a competitive optimization approach. In the proposed SSEO, adaptive inertia weight factors are employed to enhance global exploration tendencies, while a spiral search mechanism is introduced to expand the search space. In the following sections, detailed descriptions of the two strategies utilized in this study are presented.

The introduction of customized strategies in SSEO addresses the limitations of EO, resulting in a more robust optimization solution. By integrating adaptive inertia weight factors and the spiral search mechanism, SSEO significantly enhances its global exploration capabilities and expands the search space. These improvements provide SSEO with a competitive advantage, enabling it to effectively tackle complex optimization tasks. Extensive evaluations and comparisons with state-of-the-art algorithms across diverse domains, such as numerical optimization, engineering design, and real-world applications, confirm the exceptional performance of SSEO. These experimental findings validate the reliability and efficiency of SSEO as a powerful optimization tool.

### 4.1. Adaptive Inertia Weight Strategy

The basic EO algorithm employs a simple, easy-to-implement, and effective concentration updating mechanism, which enables it to rapidly converge to the optimal or suboptimal solution when faced with simple optimization problems. However, when dealing with complex multimodal optimization problems, the algorithm often gets trapped in local optima during the concentration updating process. The main reason is that the information regarding the equilibrium candidates has not been fully utilized. Specifically, one distinctive feature of the basic EO lies in the creation of an equilibrium pool. The candidates within the equilibrium pool offer knowledge about equilibrium states and establish search patterns for particles. The equilibrium pool constructed by candidates forms a fundamental component of the EO algorithm. By fully harnessing the information stored in the equilibrium candidates, it is possible to guide particles towards more promising regions. However, in the case of the basic EO, this aspect of the process did not yield the expected results, resulting in a decline in algorithm performance. Therefore, in this study, we introduced an inertia weight factor to the equilibrium candidates, aiding them to exert a more proactive influence on particles, consequently enhancing the particles’ ability to escape local optima. The adaptive concentration update equation Equation (10), formed by incorporating the inertia weight, is employed to replace the original concentration update equation. The novel mathematical model for concentration updating is as follows:

(10) $$C=\omega C_{eq}+\left(C-C_{eq}\right)\cdot F+\frac{G}{\lambda V}\left(1-F\right)$$

where ω is the inertia weight factor. To calculate ω, the following equation is utilized:

(11) $$\omega =\left(\omega_{\max}-\omega_{\min}\right)\cdot \frac{e^{10-\mu \cdot Iter}-2}{e^{10-\mu \cdot Iter}+2}+\omega_{\max}$$

where μ is a constant, Iter is the current iteration round, and $\omega_{\max}$ and $\omega_{\min}$ represent the maximum and minimum values of the inertia weight factor, respectively.

**Figure 1:**
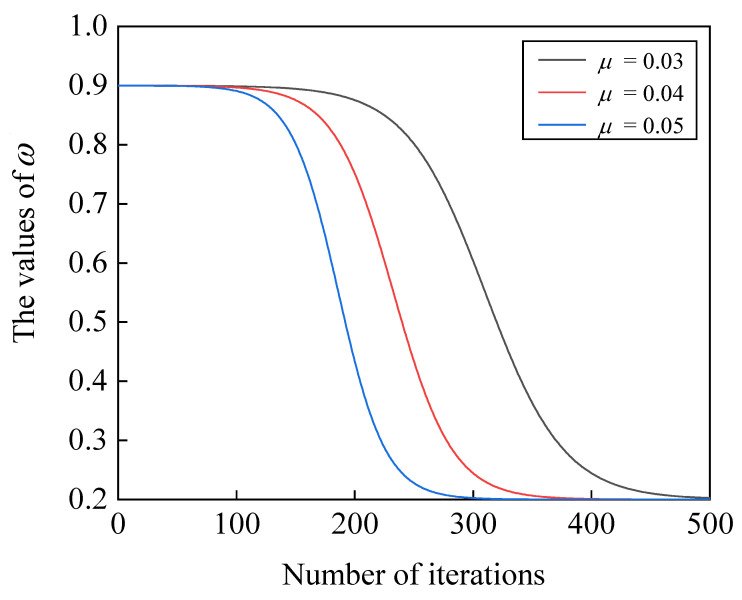
Nonlinear decay of the proposed inertia weight.

In order to visually observe the changing trend of the proposed inertia weight factor during the iterations, Figure 1 illustrates the nonlinear decay process of ω. According to Figure 1, the value of ω decreases as the iterations progress. This provides larger concentration variations to the particles in the early stages of iteration while contributing smaller concentration variations in the later stages. As a result, the algorithm is able to extensively explore the solution space during the initial iterations and finely adjust the given foreground region towards the end of the iterations.

### 4.2. Spiral Search Strategy

The EO algorithm incorporates the concept of a balance pool, which consists of five balance candidates that replace the balance state and guide the particles in their concentration updates. While this approach increases population diversity and helps the algorithm escape from local optima, it simultaneously reduces the particles’ ability to finely explore a given region. In other words, the basic EO algorithm suffers from limited local development capabilities. In order to enhance the algorithm’s local exploration ability, the spiral search mechanism is integrated into the concentration update process of the EO algorithm. This mechanism introduces a spiral movement pattern that allows particles to explore the vicinity of the current position in a more comprehensive and systematic manner. The proposed concentration update equation based on the spiral search is formulated as follows:

(12) $$C_{i}=D_{i}+e^{cl}\cdot \cos\left(2\pi \cdot rand\right)+C_{eq}$$

where $D_{i}$ denotes the distance between the current particle and the equilibrium candidate, *c* is a constant, and *l* is a random value between [0, 1]. The distance $D_{i}$ is calculated according to the following equation:

(13) $$D_{i}=abs\left(C_{eq}-C_{i}\right)$$

By incorporating the spiral search mechanism, the particles in the algorithm are guided to explore a given region with specific search behaviors. This integration effectively addresses the limitation of inadequate local exploration capability caused by the lack of particle interaction in the algorithm. Consequently, the algorithm’s local development capacity is enhanced, leading to improved solution precision. The spiral search mechanism enables the particles to efficiently delve into the local search space, allowing for finer adjustments and refinements of the solutions. As a result, the algorithm achieves higher accuracy in capturing the local optima and refining the obtained solutions.

### 4.3. The Flowchart of SSEO

**Figure 2:**
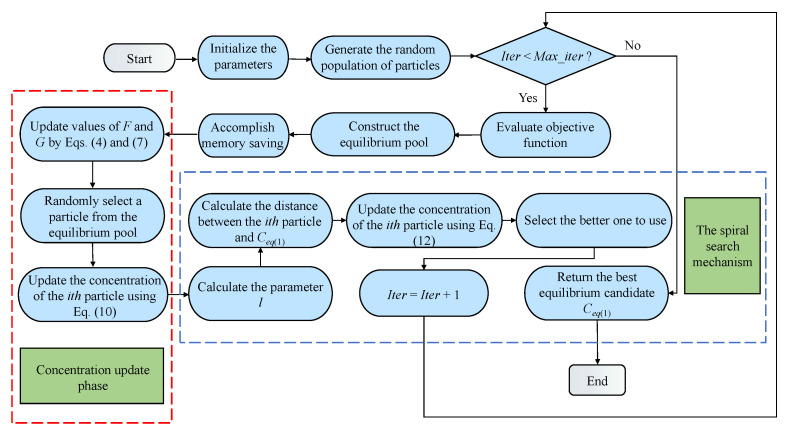
The flowchart of SSEO.

To facilitate a better understanding of the implementation details of SSEO, Figure 2 illustrates the flowchart of the SSEO. From the diagram, it can be observed that, in comparison to the basic EO algorithm, SSEO incorporates a new concentration update equation and introduces a spiral search phase.

## 5. Simulation Results and Discussion

In this section, the performance of the proposed SSEO algorithm was evaluated using the CEC2017 benchmark function set. A comprehensive comparison was conducted with the basic EO algorithm and several popular EO variants to assess the effectiveness of SSEO. The following section will provide detailed insights into the experimental setup, methodology, and analysis of the results. The experiments aimed to investigate the algorithm’s performance in terms of convergence speed, solution quality, and robustness across a diverse set of optimization problems.

### 5.1. Benchmark Functions

In this study, we conducted a performance evaluation of the algorithm using the CEC 2017 benchmark function set. The CEC 2017 test suite comprises 29 functions with diverse characteristics, which can be categorized into four types: unimodal, multimodal, hybrid, and combinatorial. The details of the benchmark functions in the CEC 2017 test suite are reported in Table 1. By executing the SSEO algorithm on these functions, the obtained results provide comprehensive insights into the algorithm’s performance. The use of the CEC 2017 test suite ensures a thorough assessment of the algorithm’s capability to handle various types of optimization problems.

### 5.2. Experimental Setup

The performance of the SSEO algorithm was compared with the basic EO and several advanced EO variants using the CEC 2017 test suite. The comparison was conducted by maintaining consistency with the specific parameters used in the respective original literature of the compared algorithms. In the SSEO algorithm, the values of $\omega_{\max}$ and $\omega_{\min}$ were set to 0.55 and 0.2, respectively. The maximum number of iterations was set to 500, and the population size was set to 30. The algorithm was executed 30 times on each function, and the mean and variance were recorded for evaluation purposes. The implementation of the algorithm was performed using MATLAB 2016b software with the utilization of the M-language. This experimental setup ensured a fair and reliable comparison of the SSEO algorithm against other algorithms in terms of their performance on the diverse functions in the CEC 2017 test suite.

### 5.3. Comparison of SSEO with Other Well-Performing EO-Based Methods

In this section, we conducted experiments using the SSEO algorithm on the 29 functions from the CEC 2017 test suite. The performance of SSEO was compared against the basic EO, recently reported EO variants, metaheuristic algorithms based on the spiral search mechanism, and other well-performing metaheuristic algorithms. The EO variants included in the comparison were mEO [41], LWMEO [43], ISEO [45], and IEO [42]. The metaheuristic algorithms based on the spiral search mechanism included MFO [48], DMMFO [49], and WEMFO [50]. Well-performing metaheuristic algorithms included PSO [8] and OOSSA [51]. Table 2 lists the parameter settings of seven algorithms. Table 3 lists the results obtained by these algorithms on 30-dimensional functions, and the statistics of the corresponding Wilcoxon signed rank tests are reported in Table 4. Table 5 lists the results obtained by these algorithms on 100-dimensional functions, and the statistics of the corresponding Wilcoxon signed rank tests are shown in Table 6. The symbols “+/=/−” in Table 4 and Table 6 represent better than, similar to, and inferior to, respectively. These tables provide a comprehensive evaluation of the algorithms’ performance and facilitate a comparative analysis of their optimization capabilities across various test functions.

**Figure 3:**
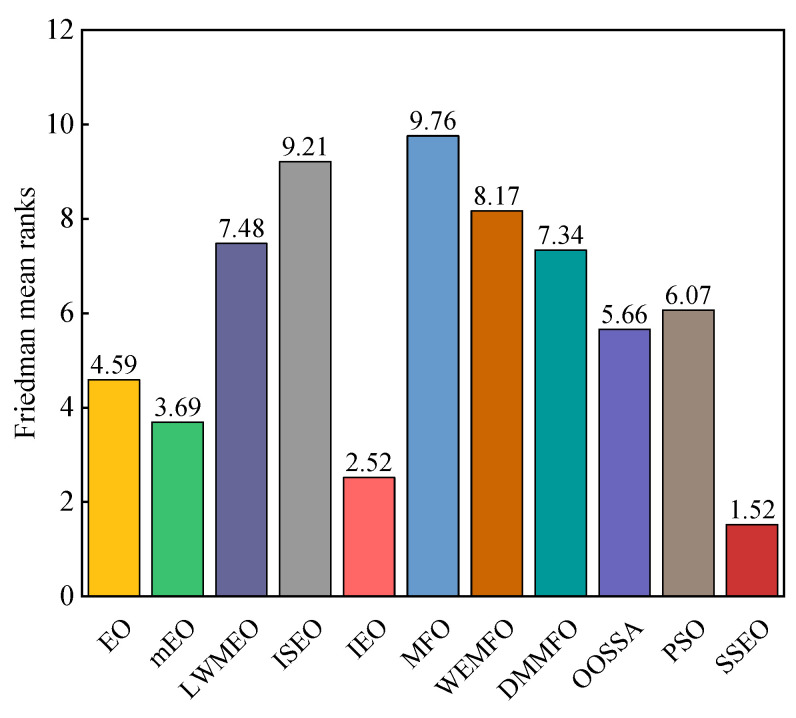
Friedman mean ranks obtained by the employed algorithms on CEC 2017 benchmark functions with 30 dimensions.

According to Table 3, the SSEO algorithm achieved the best performance in more than half of the functions. Specifically, SSEO outperformed MFO, WEMFO, DMMFO, and OOSSA on all benchmark functions. Among the 29 functions evaluated, SSEO outperformed the basic EO on 28 functions, with the exception of F23. In comparison to IEO, SSEO surpassed it on 22 functions but fell behind on 7 functions. When compared to LWMEO, SSEO outperformed it on 25 functions but was surpassed on F4 and F13. With the exception of F17, F20, and F27, SSEO demonstrated better performance than mEO on all functions. SSEO performed worse than ISEO on F27 but outperformed it on other functions. SSEO was superior to PSO on all 28 functions except F4. Additionally, Figure 3 presents the Friedman average ranking results of the algorithms on these functions. According to Figure 3, SSEO obtained the highest ranking, followed by IEO, mEO, EO, OOSSA, PSO, DMMFO, LWMEO, WEMFO, ISEO, and MFO. Moreover, according to Table 4, it is apparent that these algorithms are less than 0.05 on the vast majority of functions, which illustrates that there is a significant difference between SSEO and the other algorithms. Based on these analyses, we can conclude that the experimental results favor the performance of SSEO over the other algorithms.

**Figure 4:**
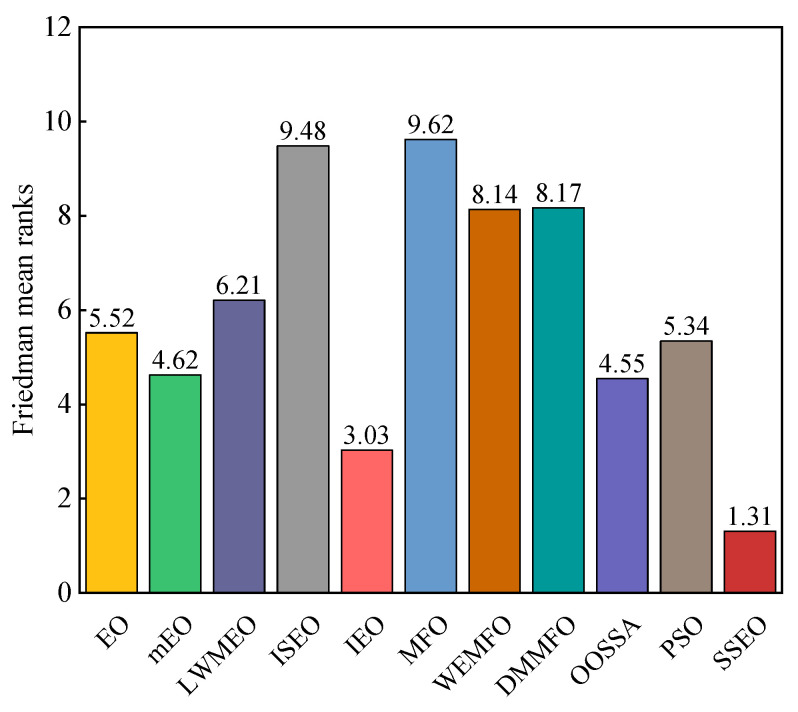
Friedman mean ranks obtained by the employed algorithms on CEC 2017 benchmark functions with 100 dimensions.

From the reported results in Table 5, it can be observed that SSEO achieved the best efficiency in most of the benchmark functions. In pairwise comparisons, SSEO outperformed the basic EO across all test cases. SSEO beat LWMEO, ISEO, MFO, WEMFO, and DMMFO across all benchmark functions. SSEO exhibited better performance than IEO in a significant number of functions and inferior performance in a few cases. Except for F11, SSEO performed better than mEO on all functions. SSEO was inferior to OOSSA in one function but surpassed it in 28 functions. Regarding PSO, SSEO was inferior to PSO on F25, while it outperformed PSO in the remaining functions. The Friedman average ranking results of these algorithms on the 29 100-dimensional functions are plotted in Figure 4. According to Figure 4, the proposed SSEO obtained the highest ranking, followed by IEO, OOSSA, mEO, PSO, EO, LWMEO, WEMFO, DMMFO, ISEO, and MFO. Furthermore, based on Table 6, the statistical analysis results of the Wilcoxon signed-rank test of these algorithms are almost lower than 0.05. This shows that there is a significant difference between the SSEO algorithm and the comparative algorithms.

Based on these analyses, we can conclude that the experimental results consistently support the superior performance of SSEO over the other algorithms.

## 6. Architecture of Mobile Robot Path Planning Using SSEO

The MRPP problem in autonomous mobile robots is a pivotal issue in robotics. This problem can be transformed into an optimization problem and solved using metaheuristic algorithms. In [52], the SSA algorithm was employed for the MRPP problem. The developed algorithm was tested in different environments, and the results showed that the algorithm was able to plan reasonable obstacle-free paths for autonomous mobile robots. In [53], a PSO-based MRPP approach was developed, and comprehensive experiments validated the effectiveness of the introduced method. In [54], a navigation strategy for a mobile robot encountering stationary obstacles was proposed using the FA algorithm. Simulation results show that the method successfully achieves the three basic objectives of path length, path smoothness, and path safety. In [55], the ABC algorithm is used in the MRPP problem to help autonomous mobile robots to generate suitable paths. The effectiveness of the algorithm is verified by simulating the algorithm under two terrains. In [56], GWO was employed for MRPP, and simulation outcomes highlighted its favorable performance in terms of path length and obstacle avoidance. In this section, we employ SSEO to address the MRPP problem and compare it with several classical metaheuristic algorithms. This evaluation aims to assess SSEO’s performance in real-world scenarios and validate its effectiveness in tackling practical optimization challenges. The empirical assessment intends to showcase SSEO’s efficacy in practical problem-solving and offer valuable insights for its practical applications.

### 6.1. Robot Path Planning Problem Description

In simple terms, the objective of the MRPP problem is to find an obstacle-free path from a starting point to a destination point. This process takes into consideration two main factors: minimizing the path length and avoiding collisions with obstacles. Based on these two factors, the following objective function has been devised:

(14) objectiveValue(OV)=L(1+σ·η)

where *L* is the path length, σ is the penalty factor, and η is a flag variable that determines whether the interpolant point is lying inside of the threatening areas. σ·η is used to determine whether the route collides with an obstacle.

The objective function of the MRPP problem is designed to balance the trade-off between finding the shortest path and ensuring obstacle avoidance. It combines the consideration of path length minimization with the incorporation of collision avoidance constraints. By formulating the problem in this way, the objective function guides the optimization algorithm, such as SSEO, to explore feasible solutions that optimize both path length and obstacle avoidance simultaneously. This formulation enables the algorithm to search for efficient and collision-free paths for the mobile robot, addressing the complexities of real-world scenarios.

### 6.2. Simulation Results

In this section, the SSEO algorithm is employed along with several well-established metaheuristic algorithms that have been validated for their performance in the MRPP problem. The MRPP problem is simulated on three different maps to evaluate the performance of these algorithms. The selected algorithms for comparison include ABC [7], PSO [8], GWO [9], FA [10], and SSA [13], which have been widely used and studied in the field. Table 7 lists the parameter settings of seven algorithms.

**Figure 5:**
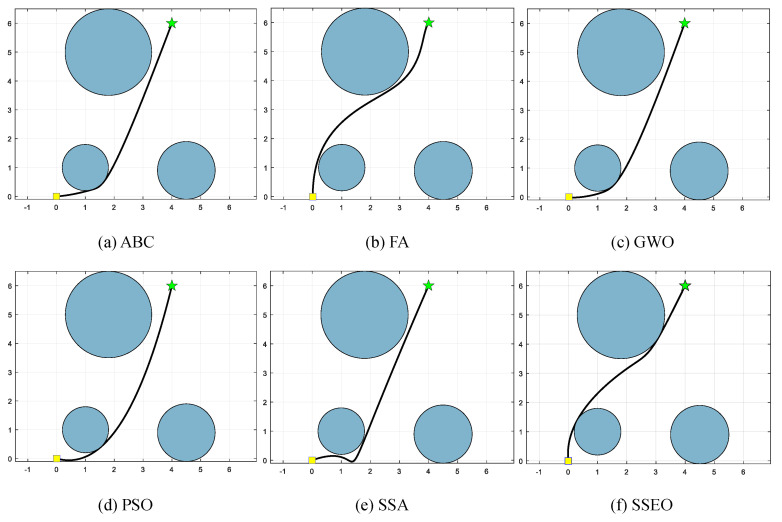
Map 1: (**a**) ABC, (**b**) FA, (**c**) GWO, (**d**) PSO, (**e**) SSA, and (**f**) SSEO.

**Figure 6:**
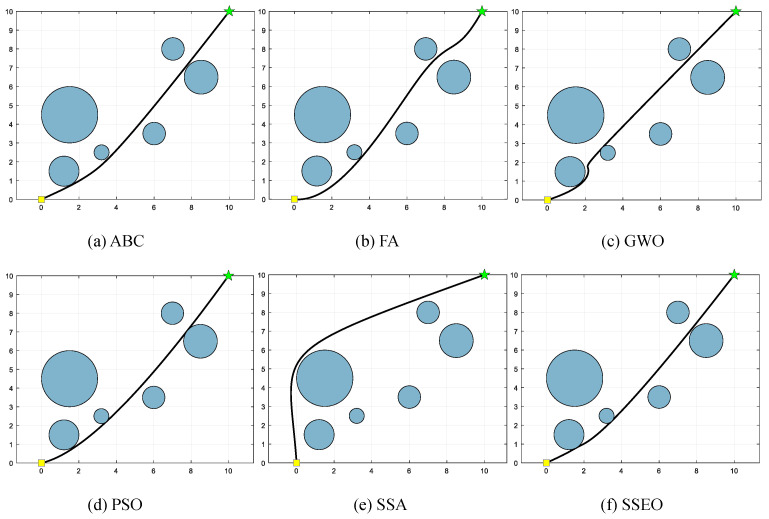
Map 2: (**a**) ABC, (**b**) FA, (**c**) GWO, (**d**) PSO, (**e**) SSA, and (**f**) SSEO.

**Figure 7:**
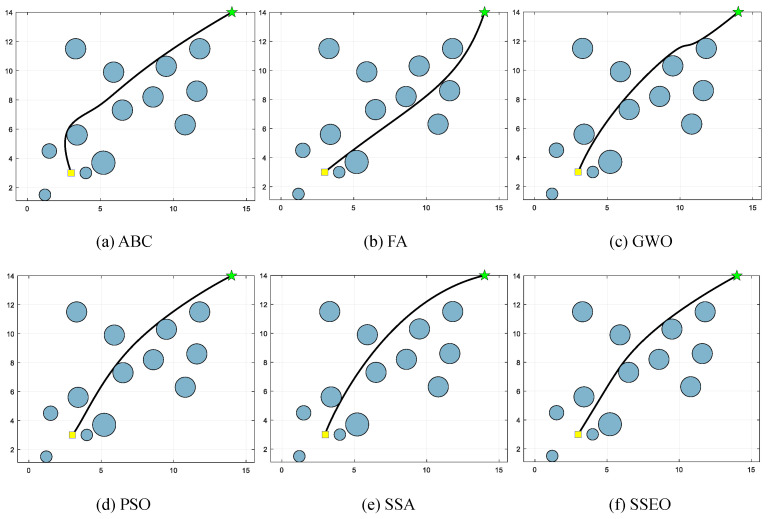
Map 3: (**a**) ABC, (**b**) FA, (**c**) GWO, (**d**) PSO, (**e**) SSA, and (**f**) SSEO.

**Figure 8:**
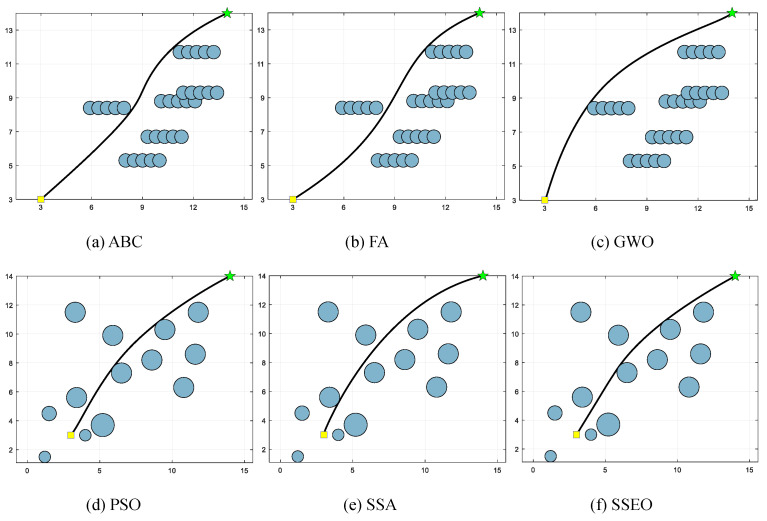
Map 4: (**a**) ABC, (**b**) FA, (**c**) GWO, (**d**) PSO, (**e**) SSA, and (**f**) SSEO.

**Figure 9:**
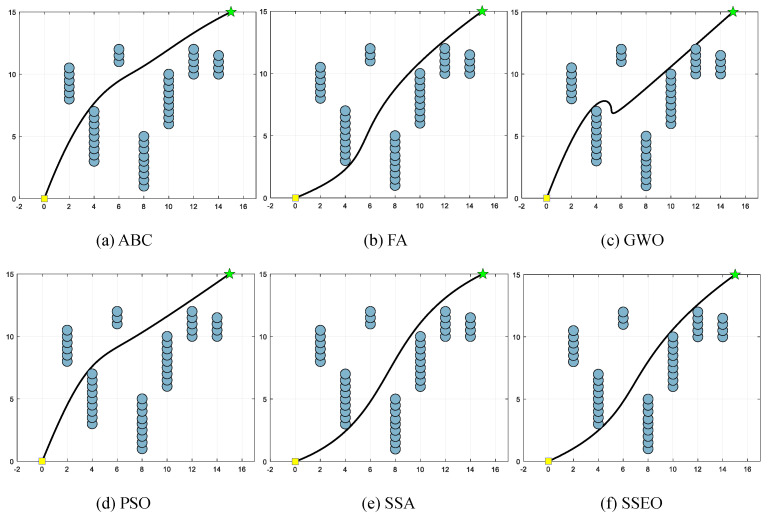
Map 5: (**a**) ABC, (**b**) FA, (**c**) GWO, (**d**) PSO, (**e**) SSA, and (**f**) SSEO.

Five environment settings, derived from [51], are chosen for simulating the MRPP problem. It should be noted that the green star indicates the end point. The details of these environment settings are presented in Table 8. The path lengths obtained by the algorithm are shown in Table 9, and the corresponding trajectories are presented in Figure 5, Figure 6, Figure 7, Figure 8 and Figure 9.

According to Table 9, the SSEO algorithm consistently produces the shortest trajectory lengths compared to the other benchmark algorithms across all environment settings. This indicates that the SSEO algorithm exhibits superior performance in solving the MRPP problem.

However, when comparing with the benchmark methods, the proposed SSEO algorithm consistently discovers shorter paths in each environment setting. This indicates that the SSEO algorithm possesses strong global optimization capabilities and the ability to avoid local optima. In summary, the SSEO algorithm shows promising potential as a path planner for the MRPP problem, outperforming the benchmark methods in terms of path length optimization and collision avoidance in diverse environments.

## 7. Conclusions

This study proposes an improved variant of EO, called SSEO, by introducing an adaptive inertia weight factor and a nature-inspired spiral search mechanism. In SSEO, the overall search framework of the basic EO is retained while incorporating the adaptive inertia weight factor and spiral search mechanism to overcome the imbalanced exploitation–exploration trade-off and suboptimal convergence performance encountered by the basic EO. The performance of the proposed SSEO algorithm is evaluated using 29 functions from the CEC 2017 benchmark test suite, and comparisons are made against the basic EO, improved EO variants, and spiral search-based metaheuristic techniques. The test results demonstrate the effectiveness of SSEO. Furthermore, the capability of the SSEO algorithm to solve real-world problems is tested using an MRPP problem. Comparative results with several classical metaheuristic algorithms reveal that SSEO is a promising path planner.

## Figures and Tables

**Table 1:** Summary of the 29 CEC 2017 benchmark problems.

Class	No.	Description	Search Range	Optimal
Unimodal	1	Shifted and Rotated Bent Cigar Function	[−100, 100]	100
	2	Shifted and Rotated Sum of Different Power Function	[−100, 100]	200
	3	Shifted and Rotated Zakharov Function	[−100, 100]	300
Multimodal	4	Shifted and Rotated Rosenbrock’s Function	[−100, 100]	400
	5	Shifted and Rotated Rastrigin’s Function	[−100, 100]	500
	6	Shifted and Rotated Expanded Scaffer’s Function	[−100, 100]	600
	7	Shifted and Rotated Lunacek Bi-Rastrigin Function	[−100, 100]	700
	8	Shifted and Rotated Non-Continuous Rastrigin’s Function	[−100, 100]	800
	9	Shifted and Rotated Levy Function	[−100, 100]	900
	10	Shifted and Rotated Schwefel’s Function	[−100, 100]	1000
Hybrid	11	Hybrid Function 1 (N = 3)	[−100, 100]	1100
	12	Hybrid Function 2 (N = 3)	[−100, 100]	1200
	13	Hybrid Function 3 (N = 3)	[−100, 100]	1300
	14	Hybrid Function 4 (N = 4)	[−100, 100]	1400
	15	Hybrid Function 5 (N = 4)	[−100, 100]	1500
	16	Hybrid Function 6 (N = 4)	[−100, 100]	1600
	17	Hybrid Function 6 (N = 5)	[−100, 100]	1700
	18	Hybrid Function 6 (N = 5)	[−100, 100]	1800
	19	Hybrid Function 6 (N = 5)	[−100, 100]	1900
	20	Hybrid Function 6 (N = 6)	[−100, 100]	2000
Composition	21	Composition Function 1 (N = 3)	[−100, 100]	2100
	22	Composition Function 2 (N = 3)	[−100, 100]	2200
	23	Composition Function 3 (N = 4)	[−100, 100]	2300
	24	Composition Function 4 (N = 4)	[−100, 100]	2400
	25	Composition Function 5 (N = 5)	[−100, 100]	2500
	26	Composition Function 6 (N = 5)	[−100, 100]	2600
	27	Composition Function 7 (N = 6)	[−100, 100]	2700
	28	Composition Function 8 (N = 6)	[−100, 100]	2800
	29	Composition Function 9 (N = 3)	[−100, 100]	2900
	30	Composition Function 10 (N = 3)	[−100, 100]	3000

**Table 2:** Parameter setting of the ten algorithms.

Algorithms	Parameters Setting
EO [31]	$a_{1}$ = 2, $a_{2}$ = 1, GP = 0.5 (Default)
mEO [39]	$a_{1}$ = 2, $a_{2}$ = 1, GP = 0.5 (Default)
LWMEO [41]	$a_{1}$ = 2, $a_{2}$ = 1, GP = 0.5, *c* = 1 (Default)
ISEO [43]	$a_{1}$ = 2, $a_{2}$ = 1, GP = 0.5 (Default)
IEO [40]	$a_{1}$ = 2, $a_{2}$ = 1 (Default)
MFO [46]	*b* = 1 and a decreases linearly from −1 to −2 (Default)
DMMFO [47]	*b* = 1 and a decreases linearly from −1 to −2 (Default)
WEMFO [48]	*b* = 1, *s* = 0, and a decreases linearly from −1 to −2 (Default)
PSO [8]	$c_{1}$ = 2, $c_{2}$ = 2, and ω linear reduction from 0.9 to 0.1 (Default)
OOSSA [51]	*b* = 0.55, *k* = 10,000, $c_{1}$ decreases nonlinearly from 2 to 0 (Default)

**Table 3:** Comparisons of eleven algorithms on CEC 2017 benchmark functions with 30 dimensions.

Function	Results	EO	mEO	LWMEO	ISEO	IEO	MFO	WEMFO	DMMFO	OOSSA	PSO	SSEO
F1	Mean	9.85E+04	6.73E+06	9.14E+03	9.62E+09	4.91E+03	1.21E+10	1.95E+08	2.03E+08	5.81E+03	9.22E+07	4.19E+03
	Std	1.04E+05	8.77E+06	8.49E+03	2.08E+09	4.84E+03	7.54E+09	1.62E+08	2.66E+08	4.58E+03	3.55E+08	5.47E+03
	f-rank	5	6	4	10	2	11	8	9	3	7	1
F3	Mean	5.21E+04	1.28E+04	5.17E+04	1.34E+05	3.67E+04	1.83E+05	7.23E+04	1.77E+05	3.32E+04	4.29E+04	4.11E+03
	Std	1.33E+04	3.43E+03	3.46E+04	3.02E+04	1.01E+04	5.35E+04	7.48E+03	4.09E+04	9.01E+03	1.21E+04	4.96E+03
	f-rank	7	2	6	9	4	11	8	10	3	5	1
F4	Mean	5.12E+02	5.09E+02	4.85E+02	1.33E+03	5.04E+02	1.05E+03	5.75E+02	5.68E+02	5.10E+02	5.02E+02	5.02E+02
	Std	1.81E+01	2.06E+01	2.93E+01	3.18E+02	1.87E+01	6.30E+02	4.88E+01	5.91E+01	1.64E+01	2.36E+01	1.92E+02
	f-rank	7	5	1	11	4	10	9	8	6	2	3
F5	Mean	5.94E+02	5.88E+02	7.35E+02	7.36E+02	5.63E+02	7.15E+02	6.75E+02	6.59E+02	6.09E+02	6.91E+02	5.79E+02
	Std	2.28E+01	2.24E+01	5.16E+01	1.98E+01	2.03E+01	5.26E+01	5.15E+01	2.62E+01	2.76E+01	2.75E+01	2.56E+01
	f-rank	4	3	10	11	1	9	7	6	5	8	2
F6	Mean	6.02E+02	6.03E+02	6.57E+03	6.18E+02	6.01E+02	6.42E+02	6.32E+02	6.30E+02	6.27E+02	6.46E+02	6.01E+02
	Std	1.94E+00	1.73E+00	7.49E+00	4.66E+00	1.28E−01	1.19E+01	1.78E+01	1.23E+01	1.17E+01	7.39E+01	9.08E−01
	f-rank	3	4	11	5	1	9	8	7	6	10	2
F7	Mean	8.41E+02	8.24E+02	1.17E+03	1.17E+03	7.99E+02	1.21E+03	9.46E+02	9.50E+02	8.66E+02	9.01E+02	8.09E+02
	Std	2.76E+01	2.54E+01	1.19E+02	9.14E+01	1.89E+01	1.58E+02	3.79E+01	7.42E+01	2.89E+01	4.17E+01	2.61E+01
	f-rank	4	3	10	9	1	11	7	8	5	6	2
F8	Mean	8.94E+02	8.79E+02	9.80E+02	1.04E+03	8.98E+02	9.99E+02	9.99E+02	9.58E+02	9.11E+02	9.41E+02	8.77E+02
	Std	2.79E+01	1.65E+01	5.27E+01	2.33E+01	1.56E+01	3.68E+01	5.16E+01	3.36E+01	2.71E+01	2.36E+01	1.71E+01
	f-rank	3	2	8	11	4	9	10	7	5	6	1
F9	Mean	1.35E+03	1.13E+03	6.67E+03	2.09E+03	9.14E+02	7.91E+03	5.78E+03	5.11E+03	3.53E+03	4.13E+03	1.02E+03
	Std	4.98E+02	2.95E+02	2.41E+03	3.44E+02	4.23E+01	2.21E+03	3.09E+03	1.63E+03	1.42E+03	7.28E+02	1.48E+02
	f-rank	4	3	10	5	1	11	9	8	6	7	2
F10	Mean	5.72E+03	5.02E+03	5.43E+03	8.66E+03	5.21E+03	5.42E+03	5.31E+03	5.25E+03	5.02E+03	4.71E+03	4.58E+03
	Std	8.37E+02	5.78E+02	7.62E+02	2.98E+02	7.59E+02	7.29E+02	7.40E+02	6.76E+02	6.03E+02	5.23E+02	7.05E+02
	f-rank	10	3	9	11	5	8	7	6	4	2	1
F11	Mean	1.25E+03	1.26E+03	1.29E+03	2.20E+03	1.20E+03	4.87E+03	1.81E+03	4.41E+03	1.34E+03	1.23E+03	1.16E+03
	Std	4.76E+01	4.28E+01	7.13E+01	3.61E+02	4.17E+01	4.51E+03	5.47E+02	3.64E+03	7.54E+01	3.72E+01	3.42E+01
	f-rank	4	5	6	9	2	11	8	10	7	3	1
F12	Mean	1.60E+06	3.21E+06	1.05E+06	2.24E+08	4.83E+05	3.11E+08	2.33E+07	8.94E+06	1.61E+07	1.04E+06	7.84E+05
	Std	1.27E+06	1.86E+06	9.06E+05	9.39E+07	5.12E+05	5.62E+08	4.34E+07	8.53E+06	1.99E+07	5.55E+05	6.07E+05
	f-rank	5	6	4	10	1	11	9	7	8	3	2
F13	Mean	2.48E+04	9.74E+04	2.08E+04	1.94E+07	2.44E+04	1.29E+08	2.66E+06	4.89E+05	9.21E+04	1.63E+05	2.37E+04
	Std	2.67E+04	5.52E+04	1.86E+04	1.55E+07	2.30E+04	4.44E+08	7.59E+06	2.41E+06	6.92E+04	8.09E+05	2.03E+04
	f-rank	4	6	1	10	3	11	9	8	5	7	2
F14	Mean	8.36E+04	6.55E+04	8.06E+04	2.33E+05	5.04E+04	3.97E+05	7.67E+05	1.21E+06	5.07E+04	5.47E+04	2.78E+04
	Std	5.95E+04	6.29E+04	7.08E+04	1.97E+05	3.71E+04	4.75E+05	8.51E+05	1.63E+06	4.24E+04	2.55E+04	2.68E+04
	f-rank	7	5	6	8	2	9	10	11	3	4	1
F15	Mean	5.68E+03	9.74E+03	1.06E+04	3.47E+06	8.39E+03	6.86E+04	4.42E+04	1.49E+04	2.62E+04	5.62E+03	4.89E+03
	Std	4.70E+03	6.67E+03	9.37E+03	7.62E+06	8.14E+03	7.24E+04	4.65E+04	1.13E+04	1.45E+04	1.33E+04	4.00E+03
	f-rank	3	5	6	11	4	10	9	7	8	2	1
F16	Mean	2.54E+03	2.46E+03	2.97E+03	3.38E+03	2.36E+03	3.18E+03	2.99E+03	2.95E+03	2.74E+03	2.72E+03	2.35E+03
	Std	3.13E+02	2.89E+02	4.19E+02	2.52E+02	3.39E+02	4.50E+02	3.45E+02	3.06E+02	4.02E+02	2.61E+02	3.15E+02
	f-rank	4	3	8	11	2	10	9	7	6	5	1
F17	Mean	2.04E+03	1.94E+03	2.55E+03	2.44E+03	1.97E+03	2.62E+03	2.43E+03	2.30E+03	2.13E+03	2.44E+03	2.03E+03
	Std	1.71E+02	1.43E+02	2.84E+02	2.16E+02	1.62E+02	2.69E+02	2.24E+02	2.62E+02	1.71E+02	2.57E+02	1.88E+02
	f-rank	4	1	10	8	2	11	7	6	5	9	3
F18	Mean	1.39E+06	4.84E+05	3.88E+05	8.69E+06	5.96E+05	8.96E+06	3.99E+06	3.20E+06	7.80E+05	8.14E+05	3.26E+05
	Std	1.62E+06	3.94E+05	3.02E+05	5.34E+06	4.77E+05	1.09E+07	3.24E+06	5.04E+06	6.95E+05	3.44E+05	3.09E+05
	f-rank	7	3	2	10	4	11	9	8	5	6	1
F19	Mean	1.30E+04	8.25E+03	1.16E+04	7.97E+05	1.09E+04	6.85E+06	2.32E+05	3.37E+04	1.93E+06	7.73E+03	6.50E+03
	Std	1.61E+04	7.86E+03	1.10E+04	9.25E+05	1.32E+04	1.94E+07	4.72E+05	5.33E+04	1.65E+06	1.12E+04	4.45E+03
	f-rank	6	3	5	9	4	11	8	7	10	2	1
F20	Mean	2.35E+03	2.25E+03	2.79E+03	2.74E+03	2.33E+03	2.74E+03	2.64E+03	2.52E+03	2.48E+03	2.67E+03	2.31E+03
	Std	1.41E+02	1.12E+02	2.81E+02	1.73E+02	1.41E+02	2.39E+02	1.96E+02	2.22E+02	1.82E+02	1.83E+02	1.43E+02
	f-rank	4	1	11	9	3	10	7	6	5	8	2
F21	Mean	2.39E+03	2.38E+03	2.52E+03	2.52E+03	2.36E+03	2.49E+03	2.46E+03	2.45E+03	2.41E+03	2.50E+03	2.35E+03
	Std	3.19E+01	2.68E+01	6.87E+01	1.38E+01	1.88E+01	4.56E+01	5.82E+1	4.94E+01	2.83E+01	3.51E+01	1.82E+01
	f-rank	4	3	11	10	2	8	7	6	5	9	1
F22	Mean	4.33E+03	2.32E+03	6.14E+03	6.35E+03	3.44E+03	6.83E+03	5.95E+03	5.05E+03	2.31E+03	4.83E+03	2.30E+03
	Std	2.24E+03	6.81E+00	2.11E+03	3.33E+03	1.98E+03	1.35E+03	2.12E+03	2.23E+03	1.17E+00	1.97E+03	1.52E+00
	f-rank	5	3	9	10	4	11	8	7	2	6	1
F23	Mean	2.73E+03	2.74E+03	2.98E+03	2.86E+03	2.71E+03	2.85E+03	2.81E+03	2.78E+03	2.78E+03	3.23E+03	2.73E+03
	Std	2.24E+01	3.17E+01	9.47E+01	1.45E+01	2.03E+01	4.64E+01	4.57E+01	3.47E+01	4.08E+01	1.17E+02	2.56E+01
	f-rank	2	4	10	9	1	8	7	5	6	11	3
F24	Mean	2.90E+03	2.90E+03	3.15E+03	3.03E+03	2.88E+03	2.98E+03	2.97E+03	2.96E+03	2.92E+03	3.25E+03	2.88E+03
	Std	2.61E+01	3.22E+01	8.49E+01	1.51E+01	2.72E+01	3.31E+01	3.19E+01	4.39E+01	3.19E+01	8.19E+01	2.08E+01
	f-rank	3	4	10	9	2	8	7	6	5	11	1
F25	Mean	2.91E+03	2.91E+03	2.92E+03	3.29E+03	2.90E+03	3.51E+03	2.96E+03	2.97E+03	2.92E+03	2.90E+03	2.89E+03
	Std	1.99E+01	1.91E+01	2.52E+01	1.39E+02	6.12E+00	7.33E+02	2.70E+01	7.55E+01	2.01E+01	1.05E+01	1.08E+01
	f-rank	5	4	7	10	2	11	8	9	6	3	1
F26	Mean	4.29E+03	4.30E+03	7.25E+03	5.92E+03	4.04E+03	5.82E+03	5.51E+03	5.45E+03	4.58E+03	5.03E+03	3.88E+03
	Std	5.61E+02	3.56E+02	1.35E+03	1.95E+02	3.71E+02	5.03E+02	4.84E+02	5.26E+02	7.29E+02	1.75E+03	6.59E+02
	f-rank	3	4	11	10	2	9	8	7	5	6	1
F27	Mean	3.23E+03	3.22E+03	3.28E+03	3.22E+03	3.22E+03	3.25E+03	3.26E+03	3.24E+03	3.24E+03	3.57E+03	3.22E+03
	Std	9.55E+01	1.01E+01	3.17E+01	6.93E+00	7.97E+02	2.62E+01	5.25E+01	1.53E+01	2.51E+01	1.40E+02	1.19E+01
	f-rank	5	2	10	1	4	8	9	6	7	11	3
F28	Mean	3.25E+03	3.26E+03	3.28E+03	3.54E+03	3.23E+03	4.20E+03	3.41E+03	3.43E+03	3.28E+03	3.24E+03	3.21E+03
	Std	2.33E+01	2.71E+01	1.24E+02	1.04E+02	2.11E+01	8.34E+02	8.01E+01	1.43E+02	3.87E+01	1.84E+01	1.88E+01
	f-rank	4	5	7	10	2	11	8	9	6	3	1
F29	Mean	3.78E+03	3.69E+03	4.24E+03	4.39E+03	3.66E+03	4.20E+03	4.23E+03	4.05E+03	4.06E+03	4.24E+03	3.65E+03
	Std	2.09E+02	1.91E+02	2.98E+02	2.43E+02	1.53E+02	3.18E+02	3.01E+02	2.48E+02	2.63E+02	2.31E+02	1.88E+02
	f-rank	4	3	10	11	2	7	8	5	6	9	1
F30	Mean	1.89E+04	8.42E+04	1.94E+04	3.60E+06	1.37E+04	1.09E+06	1.15E+06	1.38E+05	7.54E+06	1.98E+04	1.09E+04
	Std	1.76E+04	7.87E+04	1.06E+04	3.67E+06	9.90E+03	1.91E+06	2.86E+06	3.69E+05	6.31E+06	6.08E+03	3.91E+03
	f-rank	3	6	4	10	2	8	9	7	11	5	1
	Average f-rank	4.5862	3.6897	7.4828	9.2069	2.5172	9.7586	8.1724	7.3448	5.6552	6.0690	1.5172
	Overall f-rank	4	3	8	10	2	11	9	7	5	6	1

**Table 4:** Statistical conclusions based on Wilcoxon signed-rank test on 30-dimensional benchmark problems.

Function	EO *p*-Value	mEO *p*-Value	LWMEO *p*-Value	ISEO *p*-Value	IEO *p*-Value	MFO *p*-Value	WEMFO *p*-Value	DMMFO *p*-Value	OOSSA *p*-Value	PSO *p*-Value
F1	9.92E−11	3.02E−11	4.51E−02	3.02E−11	2.84E−01	3.02E−11	3.02E−11	3.02E−11	6.07E−11	2.59E−01
F3	3.69E−11	1.33E−10	1.11E−06	3.02E−11	3.26E−07	3.02E−11	3.02E−11	3.02E−11	3.02E−11	1.07E−09
F4	5.55E−02	1.58E−01	2.07E−02	3.02E−11	5.69E−01	2.15E−10	8.99E−11	6.01E−08	2.53E−04	9.94E−01
F5	4.86E−03	7.01E−02	3.69E−11	3.02E−11	6.67E−03	1.09E−10	3.47E−10	1.78E−10	6.28E−06	4.50E−11
F6	2.60E−05	3.50E−09	3.02E−11	3.02E−11	2.32E−06	3.02E−11	3.02E−11	3.02E−11	3.02E−11	3.02E−11
F7	1.41E−04	5.19E−02	3.02E−11	3.02E−11	9.63E−02	3.02E−11	3.02E−11	4.97E−11	3.82E−09	5.00E−09
F8	1.63E−02	7.39E−01	4.98E−11	3.02E−11	3.37E−05	3.02E−11	1.33E−10	7.39E−11	7.60E−07	1.09E−10
F9	3.56E−04	7.29E−03	3.02E−1	3.69E−11	1.41E−09	3.02E−11	3.02E−11	3.02E−11	3.69E−11	3.02E−11
F10	3.32E−06	1.03E−02	1.11E−04	3.02E−11	1.86E−03	6.77E−05	2.84E−04	1.06E−03	5.57E−03	5.49E−01
F11	7.12E−09	8.89E−10	1.78E−10	3.02E−11	4.46E−04	3.02E−11	3.02E−11	3.02E−11	3.02E−11	1.60E−07
F12	6.10E−03	9.83E−08	3.48E−01	3.02E−11	1.27E−02	3.02E−11	3.02E−11	5.09E−08	3.02E−11	5.40E−01
F13	7.28E−01	2.67E−09	4.55E−01	3.02E−11	1.37E−01	4.18E−09	2.78E−07	5.75E−02	1.86E−06	1.54E−01
F14	4.35E−05	3.85E−03	9.79E−05	1.69E−09	4.43E−03	3.92E−09	1.09E−10	1.01E−08	1.39E−06	1.58E−01
F15	3.71E−01	2.25E−04	2.62E−03	3.02E−11	1.15E−01	2.87E−10	3.65E−08	1.49E−04	3.50E−09	4.20E−01
F16	3.27E−02	2.12E−01	2.57E−07	5.49E−11	7.85E−01	3.82E−09	6.53E−08	6.01E−08	7.38E−10	1.64E−05
F17	9.23E−01	3.39E−02	1.56E−08	4.31E−08	2.32E−02	1.17E−09	6.53E−08	1.68E−04	9.51E−06	2.57E−07
F18	5.09E−06	3.27E−02	1.02E−01	3.02E−11	5.57E−03	1.17E−09	3.20E−09	1.55E−09	4.11E−07	1.17E−03
F19	7.62E−01	8.07E−01	6.57E−02	3.02E−11	7.51E−01	6.01E−08	9.83E−08	4.86E−03	3.69E−11	3.11E−01
F20	1.62E−01	1.30E−01	4.57E−09	8.89E−10	5.59E−01	9.26E−09	3.08E−08	1.89E−04	7.09E−08	5.46E−09
F21	8.15E−05	8.56E−04	3.02E−11	3.02E−11	8.77E−01	3.02E−11	6.70E−11	6.70E−11	1.43E−08	3.02E−11
F22	4.18E−09	3.34E−11	3.50E−09	3.02E−11	1.58E−04	3.02E−11	3.02E−11	3.02E−11	8.89E−10	3.08E−08
F23	8.65E−01	6.41E−01	3.02E−11	3.02E−11	2.50E−03	5.49E−11	8.89E−10	3.96E−08	1.07E−07	3.02E−11
F24	1.54E−01	5.90E−01	3.02E−11	3.02E−11	8.12E−04	1.09E−10	4.20E−10	1.41E−09	4.12E−06	3.02E−11
F25	7.74E−06	2.68E−06	1.29E−06	3.02E−11	3.40E−01	8.15E−11	4.98E−11	5.07E−10	5.49E−11	5.08E−03
F26	4.43E−03	6.67E−03	2.92E−09	3.02E−11	7.39E−01	3.02E−11	3.02E−11	4.50E−11	2.38E−07	1.26E−01
F27	2.71E−01	8.30E−01	1.61E−10	4.73E−01	1.99E−02	4.44E−07	1.56E−08	4.64E−05	1.55E−09	3.02E−11
F28	2.88E−06	8.35E−08	3.32E−06	3.02E−11	3.67E−03	3.02E−11	3.02E−11	3.02E−11	1.33E−10	6.55E−04
F29	2.61E−02	4.64E−01	2.03E−09	4.98E−11	9.35E−01	8.48E−09	2.92E−09	8.35E−08	2.87E−10	1.55E−09
F30	1.27E−02	6.70E−11	2.39E−04	3.02E−11	3.79E−01	3.02E−11	9.92E−11	6.70E−11	3.02E−11	1.34E−05
+/=/−	24/4/1	24/4/1	29/0/0	29/0/0	23/6/0	29/0/0	29/0/0	29/0/0	29/0/0	25/3/1

**Table 5:** Comparisons of eleven algorithms on CEC 2017 benchmark functions with 100 dimensions.

Function	Results	EO	mEO	LWMEO	ISEO	IEO	MFO	WEMFO	DMMFO	OOSSA	PSO	SSEO
F1	Mean	1.49E+10	9.12E+09	7.13E+09	1.89E+11	2.41E+09	1.57E+11	5.47E+10	5.08E+10	2.67E+09	2.81E+09	1.86E+05
	Std	5.52E+09	2.89E+09	5.39E+09	2.53E+10	2.46E+09	5.37E+10	1.00E+10	1.05E+10	7.71E+08	2.37E+09	1.29E+07
	f-rank	7	6	5	11	2	10	9	8	3	4	1
F3	Mean	5.80E+05	3.22E+05	6.83E+05	1.10E+06	5.23E+05	1.02E+06	4.11E+05	9.27E+05	2.99E+05	4.79E+05	2.86E+05
	Std	1.26E+05	3.02E+04	1.45E+05	2.93E+05	7.07E+04	1.53E+09	9.33E+04	1.40E+05	1.25E+04	9.35E+04	1.85E+04
	f-rank	7	3	8	11	6	10	4	9	2	5	1
F4	Mean	1.84E+03	1.82E+03	2.08E+03	3.83E+04	1.14E+03	2.84E+04	6.21E+03	6.50E+03	1.31E+03	1.01E+03	9.01E+02
	Std	4.12E+02	2.78E+02	5.81E+02	8.17E+03	1.31E+02	1.25E+04	1.53E+03	1.95E+03	1.14E+02	2.79E+02	6.41E+01
	f-rank	6	5	7	11	3	10	8	9	4	2	1
F5	Mean	1.32E+03	1.25E+03	1.49E+03	1.71E+03	1.10E+03	1.94E+03	1.50E+03	1.72E+03	1.14E+03	1.28E+03	1.08E+03
	Std	9.93E+01	7.06E+01	1.54E+02	7.26E+01	8.52E+01	1.96E+02	1.08E+02	1.29E+02	1.12E+02	6.47E+01	7.43E+01
	f-rank	6	4	7	9	2	11	8	10	3	5	1
F6	Mean	6.36E+02	6.36E+02	6.67E+02	6.61E+02	6.18E+02	6.84E+02	6.85E+02	6.79E+02	6.48E+02	6.63E+02	6.15E+02
	Std	6.75E+00	6.94E+00	6.51E+00	5.77E+00	3.32E+00	7.96E+00	1.37E+01	9.20E+00	2.98E+00	4.09E+00	5.60E+00
	f-rank	3	4	8	6	2	10	11	9	5	7	1
F7	Mean	2.11E+03	2.06E+03	3.58E+03	3.25E+03	1.72E+03	5.78E+03	2.86E+03	4.33E+03	1.69E+03	2.05E+03	1.71E+03
	Std	2.09E+02	1.58E+02	4.59E+02	8.75E+02	1.38E+02	6.91E+02	1.54E+02	5.24E+02	1.28E+02	2.74E+02	1.93E+02
	f-rank	6	5	9	8	3	11	7	10	1	4	2
F8	Mean	1.59E+03	1.51E+03	1.90E+03	2.00E+03	1.41E+03	2.31E+03	1.81E+03	2.04E+03	1.41E+03	1.68E+03	1.33E+03
	Std	8.92E+01	8.48E+01	2.62E+02	5.07E+01	8.38E+01	1.95E+02	1.41E+02	1.42E+02	1.12E+02	7.82E+01	1.02E+02
	f-rank	5	4	8	9	2	11	7	10	3	6	1
F9	Mean	3.52E+04	2.53E+04	3.13E+04	3.03E+04	1.85E+04	5.88E+04	5.83E+04	5.62E+04	3.34E+04	5.08E+04	2.31E+04
	Std	6.78E+03	6.39E+03	9.06E+03	5.16E+03	4.73E+03	7.54E+03	1.73E+04	1.16E+04	2.64E+03	1.15E+04	3.24E+03
	f-rank	7	3	5	4	1	11	10	9	6	8	2
F10	Mean	2.37E+04	2.12E+04	1.71E+04	3.28E+04	2.27E+04	1.98E+04	2.10E+04	1.97E+04	1.73E+04	1.59E+04	1.57E+04
	Std	1.91E+03	2.13E+03	2.22E+03	7.39E+02	1.78E+03	1.87E+03	1.38E+03	1.18E+03	1.30E+03	1.28E+03	1.38E+03
	f-rank	10	8	3	11	9	6	7	5	4	2	1
F11	Mean	6.81E+04	2.05E+04	3.48E+04	2.05E+05	4.50E+04	2.15E+05	1.09E+05	2.05E+05	4.66E+04	3.75E+04	2.66E+04
	Std	1.56E+04	4.72E+03	2.84E+04	4.29E+04	9.96E+03	5.41E+04	1.94E+04	4.52E+04	1.06E+04	1.14E+04	6.41E+03
	f-rank	7	1	3	9	5	11	8	10	6	4	2
F12	Mean	4.80E+08	7.42E+08	2.68E+08	4.90E+10	9.37E+07	4.36E+10	5.24E+09	5.97E+09	6.34E+08	1.14E+09	5.17E+07
	Std	4.79E+08	2.41E+08	1.85E+08	1.02E+10	3.81E+07	1.86E+10	1.63E+09	2.51E+09	2.29E+08	1.25E+09	2.16E+07
	f-rank	4	6	3	11	2	10	8	9	5	7	1
F13	Mean	1.23E+05	3.35E+06	1.74E+05	8.09E+09	1.62E+04	6.32E+09	8.05E+07	6.26E+07	5.27E+04	2.02E+07	4.76E+04
	Std	8.11E+04	3.01E+06	7.79E+05	1.68E+09	4.97E+03	3.68E+09	8.32E+07	8.34E+07	2.32E+04	8.85E+07	7.38E+04
	f-rank	4	6	5	11	1	10	9	8	3	7	2
F14	Mean	5.17E+06	3.81E+06	1.86E+06	6.44E+07	4.91E+06	1.87E+07	1.95E+07	1.95E+07	3.69E+06	1.90E+06	1.16E+06
	Std	2.36E+06	1.69E+06	1.01E+06	2.46E+07	2.76E+06	1.61E+07	9.59E+06	1.13E+07	1.78E+06	6.77E+05	5.22E+05
	f-rank	7	5	2	11	6	8	9	10	4	3	1
F15	Mean	2.33E+04	1.78E+05	1.67E+04	1.54E+09	5.60E+03	1.29E+09	1.53E+07	5.34E+06	5.68E+04	1.74E+04	9.29E+03
	Std	1.36E+04	1.32E+05	1.47E+04	6.20E+08	3.11E+03	1.54E+09	4.13E+07	1.18E+07	2.69E+04	9.55E+03	3.35E+03
	f-rank	5	7	3	11	1	10	9	8	6	4	2
F16	Mean	6.91E+03	7.01E+03	6.72E+03	1.07E+04	5.96E+03	8.44E+03	8.19E+03	7.37E+03	6.59E+03	6.07E+03	5.62E+03
	Std	9.98E+02	8.66E+02	8.50E+02	4.48E+02	8.09E+02	1.05E+03	9.34E+02	6.52E+02	6.09E+02	6.35E+02	6.12E+02
	f-rank	6	7	5	11	2	10	9	8	4	3	1
F17	Mean	5.24E+03	5.42E+03	6.43E+03	9.47E+03	4.97E+03	1.14E+04	6.68E+03	6.98E+03	5.49E+03	5.35E+03	5.17E+03
	Std	7.07E+02	5.65E+02	7.32E+02	9.83E+02	5.21E+02	9.67E+03	5.94E+02	9.38E+02	5.22E+02	5.66E+02	5.69E+02
	f-rank	3	5	7	10	1	11	8	9	6	4	2
F18	Mean	5.15E+06	4.25E+06	2.83E+06	1.14E+08	5.46E+06	2.46E+07	1.91E+07	2.71E+07	5.01E+06	3.49E+06	2.52E+06
	Std	2.78E+06	1.96E+06	1.39E+06	5.29E+07	2.38E+06	1.92E+07	7.92E+06	1.19E+07	3.84E+06	2.16E+06	7.85E+05
	f-rank	6	4	2	11	7	9	8	10	5	3	1
F19	Mean	7.77E+04	2.00E+06	2.62E+04	1.38E+09	4.81E+03	1.47E+09	1.50E+07	1.01E+07	6.98E+06	2.78E+06	6.60E+03
	Std	2.00E+05	1.47E+06	3.14E+04	4.65E+08	2.71E+03	1.93E+09	1.41E+07	2.49E+02	9.22E+06	1.49E+07	5.41E+03
	f-rank	4	5	3	10	1	11	9	8	7	6	2
F20	Mean	5.74E+03	5.45E+03	5.84E+03	7.78E+03	5.33E+03	5.97E+03	6.04E+03	5.76E+03	5.23E+03	5.24E+03	4.82E+03
	Std	5.75E+02	4.80E+02	4.42E+02	3.52E+02	5.38E+02	5.75E+02	5.15E+02	6.17E+02	4.97E+02	6.12E+02	6.05E+02
	f-rank	6	5	8	11	4	9	10	7	2	3	1
F21	Mean	3.01E+03	2.94E+03	3.90E+03	3.45E+03	2.83E+03	3.77E+03	3.39E+03	3.55E+03	3.08E+03	3.72E+03	2.73E+03
	Std	1.13E+02	8.88E+01	2.11E+02	5.35E+01	8.33E+01	1.35E+02	1.23E+02	1.62E+02	9.78E+01	1.17E+02	6.98E+01
	f-rank	4	3	11	7	2	10	6	8	5	9	1
F22	Mean	2.63E+04	2.36E+04	2.08E+04	3.52E+04	2.61E+04	2.18E+04	2.36E+04	2.22E+04	1.69E+04	1.89E+04	1.50E+04
	Std	1.66E+03	1.64E+03	2.12E+03	5.94E+02	2.19E+03	1.61E+03	1.53E+03	1.51E+03	7.95E+03	1.40E+03	7.27E+03
	f-rank	10	8	4	11	9	5	7	6	2	3	1
F23	Mean	3.40E+03	3.36E+03	4.63E+03	3.83E+03	3.24E+03	3.89E+03	3.83E+03	3.79E+03	3.59E+03	5.30E+03	3.21E+03
	Std	8.37E+01	8.86E+01	2.72E+02	5.35E+01	6.27E+01	1.06E+02	1.10E+02	1.54E+02	1.15E+02	3.94E+02	9.11E+01
	f-rank	4	3	10	7	2	9	8	6	5	11	1
F24	Mean	3.91E+03	3.87E+03	5.59E+03	4.33E+03	3.74E+03	4.55E+03	4.55E+03	4.39E+03	3.98E+03	5.47E+03	3.69E+03
	Std	1.13E+02	1.16E+02	3.95E+02	5.21E+01	7.83E+01	1.59E+02	2.18E+02	1.42E+02	7.98E+01	3.21E+02	1.06E+02
	f-rank	4	3	11	6	2	8	9	7	5	10	1
F25	Mean	4.49E+03	4.44E+03	4.4E+03	2.38E+04	3.83E+03	2.11E+04	7.97E+03	1.11E+04	4.23E+03	3.49E+03	3.62E+03
	Std	3.09E+02	1.84E+02	3.11E+02	5.35E+03	9.48E+01	8.01E+03	9.31E+02	2.51E+03	1.97E+02	6.99E+01	7.34E+01
	f-rank	7	6	5	11	3	10	8	9	4	1	2
F26	Mean	1.48E+04	1.31E+04	2.71E+04	1.81E+04	1.17E+04	2.01E+04	1.91E+04	1.86E+04	1.41E+04	2.01E+04	1.11E+04
	Std	2.36E+03	1.89E+03	3.15E+03	6.58E+02	2.09E+03	1.81E+03	2.01E+03	1.47E+03	1.35E+03	7.55E+03	4.72E+03
	f-rank	5	3	11	6	2	9	8	7	4	10	1
F27	Mean	3.68E+03	3.66E+03	4.19E+03	4.09E+03	3.57E+03	4.11E+03	4.09E+03	3.94E+03	3.83E+03	4.33E+03	3.55E+03
	Std	7.95E+01	9.81E+01	1.97E+02	1.92E+02	7.86E+01	2.78E+02	1.81E+02	1.39E+02	1.07E+02	3.05E+02	6.27E+01
	f-rank	4	3	10	8	2	9	7	6	5	11	1
F28	Mean	5.38E+03	4.87E+03	5.83E+03	2.05E+04	4.16E+03	1.99E+04	1.75E+04	1.69E+04	5.23E+03	3.76E+03	3.71E+03
	Std	5.75E+02	3.67E+02	1.13E+03	2.57E+03	2.24E+02	1.76E+03	3.37E+03	2.45E+03	4.82E+02	3.99E+02	6.81E+01
	f-rank	6	4	7	11	3	10	9	8	5	2	1
F29	Mean	7.75E+03	7.63E+03	8.76E+03	1.36E+04	6.81E+03	1.16E+04	9.52E+03	9.19E+03	9.79E+03	8.34E+03	6.86E+03
	Std	6.09E+02	5.53E+02	6.40E+02	1.42E+03	6.22E+02	3.28E+03	7.00E+02	8.79E+02	1.09E+03	5.81E+02	7.36E+02
	f-rank	4	3	6	11	1	10	8	7	9	5	2
F30	Mean	2.05E+06	1.38E+07	3.05E+06	3.11E+09	2.25E+05	2.94E+09	7.68E+07	6.89E+07	1.16E+08	3.95E+07	1.88E+05
	Std	1.22E+06	9.22E+06	2.54E+06	8.63E+08	1.21E+05	1.95E+09	4.32E+07	8.47E+07	7.68E+07	1.22E+08	1.23E+05
	f-rank	3	5	4	11	2	10	8	7	9	6	1
	Average f-rank	5.5172	4.6207	6.2069	9.4828	3.0345	9.6207	8.1379	8.1724	4.5517	5.3448	1.3103
	Overall f-rank	6	4	7	10	2	11	8	9	3	5	1

**Table 6:** Statistical conclusions based on Wilcoxon signed-rank test on 100-dimensional benchmark problems.

Function	EO *p*-Value	mEO *p*-Value	LWMEO *p*-Value	ISEO *p*-Value	IEO *p*-Value	MFO *p*-Value	WEMFO *p*-Value	DMMFO *p*-Value	OOSSA *p*-Value	PSO *p*-Value
F1	3.02E−11	3.02E−11	3.02E−11	3.02E−11	3.02E−11	3.02E−11	3.02E−11	3.02E−11	3.02E−11	2.15E−10
F3	3.02E−11	3.83E−06	3.02E−11	3.02E−11	3.02E−11	3.02E−11	3.34E−11	3.02E−11	3.82E−09	3.02E−11
F4	3.02E−11	3.02E−11	3.02E−11	3.02E−11	1.96E−10	3.02E−11	3.02E−11	3.02E−11	3.02E−11	9.47E−01
F5	1.61E−10	1.86E−09	3.02E−11	3.02E−11	5.59E−01	3.02E−11	3.02E−11	3.02E−11	9.26E−09	8.15E−11
F6	4.44E−07	1.03E−06	3.02E−11	3.02E−11	2.67E−09	3.02E−11	3.02E−11	3.02E−11	3.02E−11	3.02E−11
F7	3.08E−08	5.09E−08	3.02E−11	4.50E−11	6.74E−01	3.02E−11	3.02E−11	3.02E−11	5.61E−05	5.60E−07
F8	6.72E−10	4.31E−08	3.69E−11	3.02E−11	1.44E−03	3.02E−11	3.69E−11	3.02E−11	8.48E−09	8.99E−11
F9	1.41E−09	2.71E−01	3.01E−07	1.73E−07	1.68E−04	3.02E−11	6.70E−11	3.34E−11	1.10E−08	3.34E−11
F10	3.02E−11	1.78E−10	1.03E−02	3.02E−11	3.34E−11	1.29E−09	5.49E−11	2.37E−10	9.92E−11	5.30E−01
F11	3.69E−11	5.27E−05	6.10E−01	3.02E−11	4.57E−09	3.02E−11	3.02E−11	3.02E−11	3.02E−11	2.59E−05
F12	3.02E−11	3.02E−11	3.20E−09	3.02E−11	4.42E−06	3.02E−11	3.02E−11	3.02E−11	3.02E−11	1.96E−10
F13	5.97E−09	3.02E−11	5.90E−01	3.02E−11	2.19E−08	3.02E−11	3.02E−11	3.02E−11	6.74E−06	2.42E−02
F14	1.61E−10	2.87E−10	4.43E−03	3.02E−11	3.82E−10	3.02E−11	3.02E−11	3.02E−11	4.98E−11	3.37E−05
F15	4.11E−07	3.02E−11	2.89E−03	3.02E−11	3.83E−05	3.02E−11	3.02E−11	3.02E−11	4.08E−11	2.60E−05
F16	1.39E−06	2.38E−07	7.73E−06	3.02E−11	1.12E−05	3.69E−11	5.49E−11	7.39E−11	3.08E−08	1.08E−02
F17	5.11E−01	1.09E−01	3.96E−08	3.02E−11	1.49E−01	3.34E−11	3.47E−10	2.92E−09	6.20E−04	3.48E−01
F18	2.32E−06	3.37E−04	6.95E−01	3.02E−11	2.03E−07	3.34E−11	3.02E−11	3.02E−11	8.35E−08	2.81E−02
F19	1.73E−06	3.02E−11	2.43E−05	3.02E−11	2.28E−01	3.02E−11	3.02E−11	3.02E−11	3.02E−11	4.35E−05
F20	3.26E−07	1.17E−04	1.85E−08	3.02E−11	4.03E−03	2.83E−08	3.20E−09	1.49E−06	4.35E−05	2.15E−02
F21	1.78E−10	9.76E−10	3.02E−11	3.02E−11	1.34E−05	3.02E−11	3.02E−11	3.02E−11	3.02E−11	3.02E−11
F22	3.69E−11	1.61E−10	1.64E−05	3.02E−11	3.69E−11	3.08E−08	1.96E−10	2.92E−09	4.11E−07	1.02E−01
F23	1.01E−08	1.25E−07	3.02E−11	3.02E−11	4.68E−02	3.02E−11	3.02E−11	3.69E−11	3.34E−11	3.02E−11
F24	6.52E−09	4.11E−07	3.02E−11	3.02E−11	2.71E−02	3.02E−11	3.02E−11	3.02E−11	3.69E−11	3.02E−11
F25	3.02E−11	3.02E−11	3.34E−11	3.02E−11	9.75E−10	3.02E−11	3.02E−11	3.02E−11	3.02E−11	1.25E−07
F26	5.61E−05	4.03E−03	4.08E−11	1.07E−07	6.31E−01	5.97E−09	2.60E−08	3.65E−08	1.29E−06	2.96E−05
F27	4.69E−08	1.25E−05	3.02E−11	3.02E−11	1.37E−01	3.34E−11	3.02E−11	4.51E−11	3.02E−11	3.02E−11
F28	3.02E−11	3.02E−11	3.02E−11	3.02E−11	3.69E−11	3.02E−11	3.02E−11	3.02E−11	3.02E−11	1.03E−02
F29	1.43E−05	4.94E−05	5.07E−10	3.02E−11	9.35E−01	3.69E−11	4.98E−11	3.16E−10	3.02E−11	9.26E−09
F30	4.08E−11	3.02E−11	4.08E−11	3.02E−11	9.05E−02	3.02E−11	3.02E−11	3.02E−11	3.02E−11	3.02E−11
+/=/−	28/1/0	29/0/0	26/3/0	29/0/0	24/4/1	29/0/0	29/0/0	29/0/0	29/0/0	27/2/0

**Table 7:** Parameter setting of the five algorithms.

Algorithms	Parameters Setting
ABC [7]	Limit = 50 (Default)
PSO [8]	$c_{1}$ = 2, $c_{2}$ = 2, and ω linear reduction from 0.9 to 0.1 (Default)
GWO [9]	*a* linear reduction from 2 to 0 (Default)
FA [10]	*g* = 1, *a* = 0.2, *r* = 0.5 (Default)
SSA [13]	$c_{1}$ decreases nonlinearly from 2 to 0 (Default)

**Table 8:** Type of environment.

Terrain	No.	Initial	Final	X Axis	Y Axis	Obstacle Radius
	**Obstacle**	**Coordinates**	**Coordinates**			
Map 1	3	0, 0	4, 6	[1 1.8 4.5]	[1 5.0 0.9]	[0.8 1.5 1]
Map 2	6	0, 0	10, 10	[1.5 8.5 3.2 6.0 1.2 7.0]	[4.5 6.5 2.5 3.5 1.5 8.0]	[1.5 0.9 0.4 0.6 0.8 0.6]
Map 3	13	3, 3	14, 14	[1.5 4.0 1.2 5.2 9.5 6.5 10.8	[4.5 3.0 1.5 3.7 10.3 7.3 6.3	[0.5 0.4 0.4 0.8 0.7 0.7 0.7 0.7
				5.9 3.4 8.6 11.6 3.3 11.8]	9.9 5.6 8.2 8.6 11.5 11.5]	0.7 0.7 0.7 0.7 0.7]
Map 4	30	3, 3	14, 14	[10.1 10.6 11.1 11.6 12.1 11.2	[8.8 8.8 8.8 8.8 8.8 11.7 11.7	[0.4 0.4 0.4 0.4 0.4 0.4 0.4 0.4
				11.7 12.2 12.7 13.2 11.4 11.9	11.7 11.7 11.7 9.3 9.3 9.3 9.3	0.4 0.4 0.4 0.4 0.4 0.4 0.4 0.4
				12.4 12.9 13.4 8 8.5 9 9.5 10	9.3 5.3 5.3 5.3 5.3 5.3 6.7 6.7	0.4 0.4 0.4 0.4 0.4 0.4 0.4 0.4
				9.3 9.8 10.3 10.8 11.3 5.9 6.4	6.7 6.7 6.7 8.4 8.4 8.4 8.4 8.4]	0.4 0.4 0.4 0.4 0.4 0.4]
				6.9 7.4 7.9]		
Map 5	45	0, 0	15, 15	[2 2 2 2 2 2 4 4 4 4 4 4 4 4 4	[8 8.5 9 9.5 10 10.5 3 3.5 4 4.5 5	[0.4 0.4 0.4 0.4 0.4 0.4 0.4 0.4
				6 6 6 8 8 8 8 8 8 8 8 8 10 10	5.5 6 6.5 7 11 11.5 12 1 1.5 2 2.5	0.4 0.4 0.4 0.4 0.4 0.4 0.4 0.4
				10 10 10 10 10 10 10 12 12	3 3.4 4 4.5 5 6 6.5 7 7.5 8 8.5 9 9.5	0.4 0.4 0.4 0.4 0.4 0.4 0.4 0.4
				12 12 12 14 14 14 14]	10 10 10.5 11 11.5 12 10 10.5 11 11.5]	0.4 0.4 0.4 0.4 0.4 0.4 0.4 0.4
						0.4 0.4 0.4 0.4 0.4 0.4 0.4 0.4
						0.4 0.4 0.4 0.4 0.4]

**Table 9:** The minimum route length comparison of SSEO-based MRPP method and comparison approaches under five environmental setups.

Terrain	PSO	FA	ABC	GWO	SSA	SSEO
	**Path Length**	**Path Length**	**Path Length**	**Path Length**	**Path Length**	**Path Length**
Map 1	7.8497	7.6093	7.7471	7.7713	8.0469	7.4575
Map 2	14.3354	14.5336	14.3881	14.4311	16.5022	14.3132
Map 3	15.8629	15.866	16.9046	15.9311	16.2811	15.8597
Map 4	16.2247	15.8489	15.7883	16.2379	16.2793	15.7398
Map 5	21.9021	21.6739	21.9537	23.3205	21.6779	21.5298

## Data Availability

The data presented in this study are available on request from the corresponding author.
